# Role of Mismatch Repair Deficiency Status and Microsatellite Instability in Relation to the Expression of Immune Checkpoint Proteins in Colorectal Cancer

**DOI:** 10.7759/cureus.43571

**Published:** 2023-08-16

**Authors:** Antariksha Sharma, Vandana Raphael, Bifica S Lyngdoh, Caleb Harris, Vikas K Jagtap

**Affiliations:** 1 Pathology, North Eastern Indira Gandhi Regional Institute of Health and Medical Sciences, Shillong, IND; 2 Pathology, All India Institute of Medical Sciences, Guwahati, IND; 3 Surgical Oncology, North Eastern Indira Gandhi Regional Institute of Health and Medical Sciences, Shillong, IND; 4 Radiation Oncology, North Eastern Indira Gandhi Regional Institute of Health and Medical Sciences, Shillong, IND

**Keywords:** immune checkpoint proteins, programmed death ligand-1, mmr status, microsatellite instability (msi), colorectal cancer

## Abstract

Introduction

Colorectal cancer (CRC) is the third most common cancer in the world among men and second among women worldwide. One of the major molecular pathways responsible for the development of colorectal cancer (CRC) is the microsatellite instability (MSI) pathway. During carcinogenesis, the tumor cells express programmed death ligand-1 (PD-L1), which reduces the immunogenicity leading to the escape of immune attack. Anti-PD-L1 interaction is an upcoming line of research for the treatment of colorectal carcinoma patients.

Materials and methods

The present study was an ambispective study where the mismatch repair deficiency status (MMR) and programmed death ligand-1 (PD-L1) expression were studied using immunohistochemistry and then later analyzed and compared with the clinicopathological parameters and MSI status in relation to the expression of programmed death ligand-1 (PD-L1) in neoplastic and immune cells in a total of 55 biopsy specimen. MMR expression was reported as retained or loss of nuclear staining, and PD-L1 expression was taken as positive with a cut-off of more than or equal to 5% membranous positivity in both tumor cells and immune cells.

Results

The analysis showed a significant correlation of microsatellite instability (MSI) status with two of the clinicopathological parameters, which were the site of the tumor (p-value<0.001) and M stage (p-value<0.001). PD-L1 expression in neoplastic cells showed no significant correlation with the clinicopathological parameters, whereas PD-L1 expression in immune cells showed a significant association with gender (p-value=0.043). Also, MSI status showed a significant association with PD-L1 expression in tumor cells (p-value <0.001).

## Introduction

Colorectal carcinoma (CRC) is an epithelial malignant tumor of the colon or rectum. Malignancy is considered when the tumor invades through the muscularis mucosae into the submucosa [1]. The incidence of colorectal cancer is 10.2%, and a mortality rate of 9.2% among all cancers in the world [2]. A total of 1.8 million new cases and a mortality rate of 0.88 million were recorded till 2018. From 1975 to 2013, CRC incidence rate increased from 10 to 15 cases per 100,000 population of Americans between the age of 20-49 years old [2]. The incidence of colorectal cancer is lower in India compared to the Western countries and is ranked ninth in India among both males and females. The age-standardized rate (ASR) for CRC in India is low at 7.2 per 100,000 population in males and 5.1 per 100,000 population in women. However, in a country with a population of a billion plus people, the absolute number of patients suffering from CRC is large. Five-year survival of CRC in India is one of the lowest in the world at less than 40% [3].

The major molecular pathways responsible for the development of colorectal cancer (CRC) are the chromosomal instability (CIN) pathway, microsatellite instability (MSI) pathway, and CpG island methylator phenotype (CIMP) pathway [4].

Microsatellite instability is a defect that is seen in any of the mismatch repair (MMR) genes (MLH1, MSH2, PMS2, GTBP/MSH6). This causes an inability to identify and repair errors that occur during DNA replication. The loss of DNA MMR movement increases the pace of apoptosis and changes related to development control which leads to the progression of an increasingly fast adenoma-to-carcinoma sequence [5].

Programmed cell death protein 1 (PD-1) is an inhibitory checkpoint molecule. It is a member of the CD28 family and is demonstrated on the surface of activated T cells to control proliferation and activation. The dominant ligand for PD-1 is PD-L1 (also known as B7-H1) and is expressed in activated T cells, B cells, dendritic cells, macrophages, endothelial cells, and a large number of tumor cells. The activation of the PD-1/PD-L1 pathway in the normal immune system inhibits the immune function of T lymphocytes and promotes the inhibitory function of regulatory T cells, which leads to a reduction of the immune response of the body. During carcinogenesis, the tumor cells express PD-L1, which causes a reduction of immunogenicity; thus, it is not recognized by the immune system and escapes the immune attack [6]. PD-L1 positivity is seen in cases with advanced pathological features, which suggests its role in the tumorigenesis of gastric and gastroesophageal junction adenocarcinoma [7].

Considering the fact that colorectal cancer is a leading cause of cancer death worldwide and patient survival depends mainly on early diagnosis, no study has been done in north-east India to correlate the expression of MSI and MMR status in relation to the immune checkpoint proteins and its clinicopathological significance.

The present study was carried out to focus on the relationship between MSI- MMR instability in relation to the immune checkpoint proteins (PD-L1) and its clinicopathological characteristics, which may help in guiding tumor-specific therapies in colorectal carcinomas.

## Materials and methods

The present study was conducted in the Department of Pathology of North Eastern Indira Gandhi Regional Institute of Health and Medical Sciences (NEIGRIHMS) from May 2020 to July 2021. It was an ambispective analytical study.

The inclusion criteria included all patients diagnosed by histopathological examination with colorectal cancer in NEIGRIHMS with effect from 2018 to 2021. The exclusion criteria included patients who had received any neoadjuvant therapy (radiotherapy and/or chemotherapy), biopsies with inadequate material, and inconclusive biopsies. All cases of colorectal cancer presenting to the NEIGRIHMS outpatient department (OPD) within a span of four years (2018-2021) were included in the study.

The relevant clinical and radiological data were obtained for all the cases diagnosed by histopathological examination as colorectal cancer. Tumors were histologically verified. The cases with prior neoadjuvant therapy were excluded. Approval for the study was granted by the Institute Research and Ethics Committee (NEIG/IEC/M7/T12/). The American Joint Committee on Cancer (AJCC) tumor, node and metastasis (TNM) classification guidelines were used for the clinical staging of the tumor. Assessment of MLH1, PMS2, MSH2, MSH6, and PD-L1 were done immunohistochemically by the Horse Radish Peroxidase (HRP) method. The paraffin-embedded sections of the tumor were stained by monoclonal antibodies raised against MLH1 (Dako, Clone ES05), PMS2 (Dako, Clone EP51), MSH6 (Dako, Clone EP49), MSH2 (Dako, Clone FE11) and PDL1 (Clone Cal 10, Master Diagnostica, Spain) according to manufacturer's instructions and scoring was done.

**Table 1:** Evaluation of IHC expression for MMR proteins IHC - immunohistochemistry; MMR - mismatch repair

MMR expression	Interpretation
Any amount of nuclear staining in the neoplastic cells	Intact/retained MMR expression
Complete loss of nuclear staining in the neoplastic cells	Loss of MMR expression

Each slide was examined under a light microscope without the knowledge of the patient's other data by two pathologists independent of each other. For internal quality control and standardization of the process, sections of normal colonic tissue and appendix were used as positive controls, and sections with absent primary antibodies as negative controls. According to the presence or absence of nuclear staining and intensity of staining of neoplastic cells, and the percentage of positive cells, the results of immunostaining in tissues were scored for MMR proteins and PD-L1. The cut-off for PD-L1 positive stained tumor is taken as membranous staining of more than or equal to 5% of tumor cells; the expression <5% was evaluated as negative. MLH1, PMS2, MSH2, and MSH6 were analyzed as a loss when there was a complete loss of nuclear stain and retained when there was any amount of intact nuclear expression. The interpretation of immunohistochemistry (IHC) for MMR proteins and PD-L1 is enumerated in Tables 1, 2.

**Table 2:** Scoring of IHC for PD-L1 expression PD-L1 - programmed death ligand 1

Score	PD-L1 membranous staining in tumor cells (A)	Staining intensity (B)
0	No cells stained	No immunostaining
1	≤10% positive cells	Weak or light yellow immunostaining
2	10-50% positive cells	Moderate brown immunostaining
3	≥50% positive cells	Strong deep brown immunostaining

The cases were classified as microsatellite stable (MSS) when all four antibodies showed positive nuclear staining of the tumor cells, as microsatellite unstable low (MSI-L) when one antibody showed negative nuclear staining of the tumor cells, and as microsatellite unstable high (MSI-H) when two antibodies or more show negative nuclear staining of the tumor cells [8-10]. 

## Results

**Figure 1:**
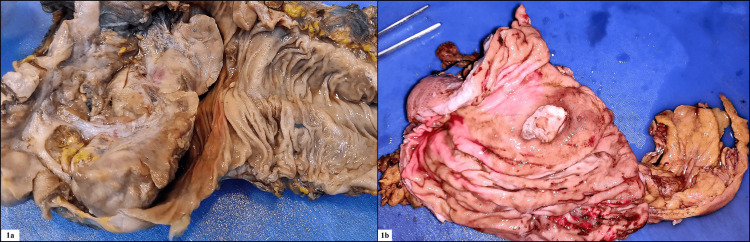
(1a) gross appearance of a proliferative lesion in a hemicolectomy specimen of adenocarcinoma colon and (1b) gross appearance of an ulcerative lesion in an abdominoperineal resection specimen of adenocarcinoma rectum

**Figure 2:**
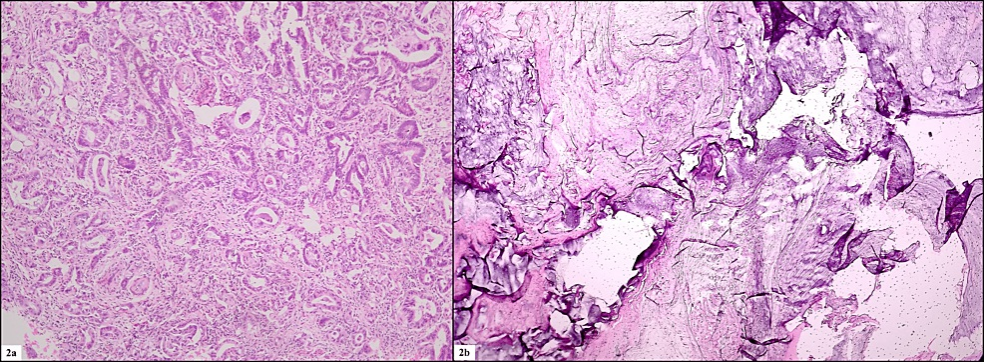
(2a) photomicrograph of a well-differentiated adenocarcinoma (H&E, 100x) and (2b) photomicrograph of m,cinous adenocarcinoma (H&E, 100x)

A total of 55 cases fulfilling our inclusion and exclusion criteria were taken for analysis. Forty-one were core biopsy specimens, 12 were hemicolectomy specimens, one was an abdominoperineal resection specimen, and one was an anterior resection specimen (Figures 1a, 1b). The age range was 18 to 85 years, the mean age was 47.18 years, and the median was 45 years. Twenty-three patients were males, and 32 patients were females, with a female-to-male ratio of 1.3:1. Most tumors were located in the rectum, which constituted 29 cases, followed by te right colon 15 cases, recto-sigmoid colon, which constituted four cases. The least common site for tumor locations were the sigmoid colon, transverse colon, and left colon, which constituted three cases, three cases, and one case, respectively. Thus, the proximal colon composed 18 cases, and the distal colon with rectum composed 37 cases. Most of the cases were moderately differentiated adenocarcinoma (MDAC) which constituted 26 cases, well-differentiated adenocarcinoma (WDAC), which constituted 14 cases, followed by mucin-secreting carcinoma, which constituted nine cases (Figures 2a, 2b). The least common types included poorly differentiated adenocarcinoma (PDAC), which constituted five cases, followed by signet ring adenocarcinoma, which constituted one case. Thus, 42 cases were of low grade, and 13 cases were of high grade.

According to the AJCC protocol for tumor staging, this study included one case of Tx, one case of T1, six cases of T2, 39 cases of T3, and eight cases of T4. Nodal staging of the tumours included 16 cases of N0, 27 cases of N1, 11 cases of N2, and one case of N3. There were nine cases of M1, and 46 cases showed no distant metastasis (M0). Thirty-two cases belonged to stage III, nine cases each belonged to stage II and stage IV, and five cases belonged to stage I.

**Figure 3:**
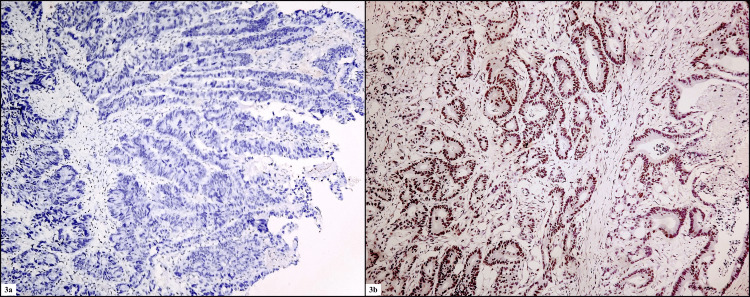
(3a) IHC photomicrograph of MMR protein loss in tumor cells (100x) and (3b) IHC photomicrograph of MMR protein retained in tumor cells (100x) IHC - immunohistochemistry; MMR protein - mismatch repair protein

The cases which showed loss of expression of MLH1 were 18.2% (n=10), 29% (n=16) cases showed loss of PMS2 expression, 3.6% (n=2) cases showed loss of MSH2 expression, and 7.2% (n=4) cases showed loss of MSH6. Thus, a total of 20% (n=11) cases showed MSI-H instability, 12.7%(n=7) cases showed MSI-L, and 67% (n=37) cases showed MSS instability (Figures 3a, 3b).

**Figure 4:**
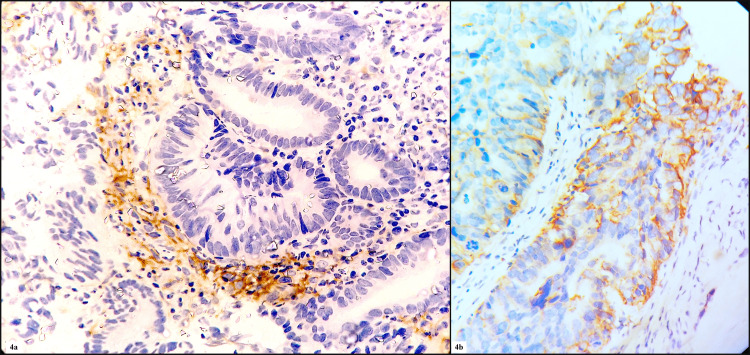
(4a) IHC photomicrograph of PD-L1 positivity in immune cells and negative in tumor cells (400x) and (4b) IHC photomicrograph of PD-L1 positivity in tumor cells and negative in immune cells (400x) IHC - immunohistochemistry; PD-L1 - programmed death ligand 1

PD-L1 positivity in neoplastic cells was seen in 30.9% (n=17) cases, and 69% (n=38) cases showed negativity for PDL1. PD-L1 positivity in immune cells was seen in 50.9% (n=28) cases, and 49% (n=27) cases showed negativity for PD-L1 (Figures 4a, 4b). The association of MSI status and PD-L1 expression in neoplastic and immune cells with age, sex, histological grading, tumor subtype, tumor site, and TNM staging are enumerated in Tables 1, 2. The association of MSI status with PD-L1 expression in tumor cells and immune cells are enumerated in Tables 3, 4, 5.

**Table 3:** Association of MSI status with clinicopathological parameters. MSI - microsatellite instability; MSI-H - microsatellite instability-high; MSI-L - microsatellite instability-low; MSS - microsatellite stable; NOS - not otherwise specified

Clinicopathological parameters		MSI-H (n=11)	MSI-L (n=07)	MSS (n=37)	p-value
Age	<50 years	05	06	20	0.230
	≥50 years	06	01	17	
Sex	Male	04	04	15	0.713
	Female	07	03	22	
Histological grading	Low grade (well-differentiated and moderately differentiated)	09	04	29	0.500
	High grade (poorly differentiated)	02	03	08	
Tumor subtype	Adenocarcinoma NOS	09	05	31	0.763
	Mucinous adenocarcinoma and Signet-ring cell carcinoma	02	02	06	
Tumor site	Distal colon and rectum	02	04	31	<0.001
	Proximal colon	09	03	06	
T staging	Tx+T1+T2	02	00	06	0.715
	T3+T4	09	07	31	
N staging	N0+N1	10	06	27	0.541
	N2+N3	01	01	10	
M staging	M0	11	02	33	<0.001
	M1	00	05	04	
Staging	Stage I+II	03	01	10	0.901
	Stage III+IV	08	06	27	

**Table 4:** Association of PD-L1 status in neoplastic cells and immune cells with clinicopathological parameters PD-L1 - programmed death ligand 1; NCs - neoplastic cells; ICs - immune cells; NOS - not otherwise specified

Clinicopathological parameters		PD-L1 negative in NCs (n=38)	PD-L1 positive in NCs (n=17)	p-value	PD-L1 negative in ICs (n=27)	PD-L1 positive in ICs (n=28)	p-value
Age	<50 years	22	09	0.732	17	14	0.333
	≥50 years	16	08		10	14	
Sex	Male	18	05	0.212	15	08	0.043
	Female	20	12		12	20	
Histological grading	Low grade (well-differentiated and moderately differentiated)	29	13	1.000	18	24	0.096
	High grade (poorly differentiated)	09	04		09	04	
Tumor type	Adenocarcinoma NOS	30	15	0.707	20	25	
	Mucinous adenocarcinoma and Signet-ring cell carcinoma	08	02		07	03	0.177
Tumor site	Distal colon	27	10	0.372	18	19	0.925
	Proximal colon	11	07		09	09	
T staging	Tx+T1+T2	04	04	0.235	03	05	0.075
	T3+T4	34	13		24	23	
N staging	N0+N1	30	13	1.000	21	22	0.943
	N2+N3	08	04		06	06	
M staging	M0	31	15	0.705	22	24	0.729
	M1	07	02		05	04	
Staging	Stage I+II	08	06	0.322	06	08	0.589
	Stage III+IV	30	11		21	20	

**Table 5:** Association of MSI status with PD-L1 expression in tumour cells and immune cells MSI - microsatellite instability; MSI-L - microsatellite instability-low; MSI-H - microsatellite instability-high; MSS - microsatellite stable; PD-L1 - programmed death ligand 1

MSI status		PD-L1 positive in tumor cells (n=17)	PD-L1 negative in tumor cells (n=38)	p-value	PD-L1 positive in immune cells (n=28)	PD-L1 negative in immune cells (n=27)	p-value
MSI-H	n=11	09	02	<0.001	06	05	0.514
MSI-L	n=7	00	07		02	05	
MSS	n=37	08	29		20	17	

## Discussion

For the present study, a total of 55 cases of colon and rectal adenocarcinoma diagnosed on histopathology that fulfilled the inclusion criteria were taken into consideration.

Colorectal cancers with MSI-H phenotype are predominantly seen in females, located mostly in the right (proximal) colon, and show a poorer differentiation or mucinous type histology. Clinically, they show a lower aggressive clinical course, seen at a lower stage, and seem to have improved sensitivity to some adjuvant treatments [5,11].

In a study done by Phipps et al., 82% of MSI-H tumours were located in the proximal colon, compared to 31% of MSS/MSI-L tumours [11,12].

A study undertaken by Kaur et al. showed 18.7% of colorectal cancers lack any one of the MMR gene proteins expression. Abnormal MMR protein expression was seen to be related to right-sided colon cancers, histological types of cancer like signet ring carcinomas and mucinous carcinomas, and histological differentiation of cancers [5,13].

In a study undertaken by Hemminki et al., 82% of the MSI unstable cases were in the proximal colon compared with 36% of the MSI stable cases (p=0.0066). Most cases were histologically moderately differentiated in both groups (64% and 69% for the MSI unstable and MSI stable groups, respectively) [14]. A study done by Samowitz et al. showed a significant correlation between microsatellite instability and proximal tumor location, female gender, young and old age at diagnosis, poor histological differentiation, and low tumor stage [15].

A study done by Yuan et al. showed tumours with MSI-H have a predilection for the right colon and display poorer differentiation. Also, MSI-H colorectal carcinoma showed lower stage in comparison with patients having MSS tumors, and dMMR tumors did not show metastasis to lymph nodes or distant organs compared to MSS ones [16].

In our study, out of the total 55 cases of colorectal cancers, 18 (32.7%) cases showed deficient MMR protein expression and 37 (67.2%) were MSS. Individual MLH1 expression was seen to be lost in 10 (18.1%) cases, whereas 45 (81.8%) cases showed retained expression of MLH1. 16 (29%) cases showed an individual lack of PMS2 expression, and the rest 39 (70.9%) cases showed intact PMS2 expression. Two (3.6%) cases showed a lack of individual MSH2 expression, and the rest 53 (96.3%) cases showed intact MSH2 expression. Four (7.2%) cases showed a lack of MSH6 expression, with the rest 51 (92.7%) cases showing an intact MSH6 expression. Together loss of both MLH1 and PMS2 was seen in 10 (18.1%) cases, and together loss of both MSH2 and MSH6 were seen in two (3.6%) cases. Loss of all four MMR protein expressions together was seen in only one (1.8%) case. Thus, 11 (20%) cases showed high microsatellite instability MSI-H, seven (12.72%) cases showed low microsatellite instability MSI-L and the rest 37 (67.2%) cases were microsatellite stable MSS. Abnormal MMR expression was seen in proximal tumours of the colon, which constituted a total of 18 (32.7%) cases in the proximal colon, and the rest 37 (67.2%) were in the distal colon and rectum, which were MSS. Nine (50%) cases belonged to MSI-H, and three (16.6%) cases belonged to MSI-L out of the total 18 cases of dMMR expression. Thus, there was a significant association of MSI status with the site of the tumor in our study (p-value<0.001) which were situated in the proximal colon. Also, out of the total 55 cases of CRCs, a total of nine (16.3%) cases showed distant metastasis and the rest 46 (83.6%) cases showed no distant metastasis. All 11 (100%) MSI-H tumor cases showed no distant metastasis (M0), whereas five (55.5%) cases out of the seven MSI-L cases showed distant metastasis, and the other four (44.4%) cases showed distant metastasis were MSS. Thus, there was a significant correlation between M staging and the MSI status (p-value<0.001), which leads to the conclusion that MSI-H tumors show a lower rate of distant metastasis. In contrast to the other studies, our study did not show any significant correlation with the other clinicopathological parameters. This may be due to a smaller sample size and type of biopsy specimen, as our highest number of cases were colonoscopic biopsies which might have led to a disparity in histological grade and subtyping of tumours.

**Table 6:** Comparison of various studies and the present study showing the correlation of different clinicopathological parameters with MSI status MSI - microsatellite instability

Studies	Age	Gender	Site	Histological grade	Histological subtype	T Stage	N Stage	M Stage	Stage
Samowitz et al. [15]	Significant	Significant	Significant	Significant	Not Significant	Significant	Not Significant	Not Significant	Not Significant
Kaur et al. [5]	Not Significant	Not Significant	Significant	Significant	Significant	Not Significant	Not Significant	Not Significant	Not Significant
Yuan et al. [16]	Not Significant	Not Significant	Significant	Not Significant	Not Significant	Not Significant	Not Significant	Significant	Significant
Hemminki et al. [14]	Not Significant	Not Significant	Significant	Not Significant	Not Significant	Not Significant	Not Significant	Not Significant	Not Significant
Phipps et al. [11]	Not Significant	Not Significant	Significant	Not Significant	Not Significant	Not Significant	Not Significant	Not Significant	Not Significant
Our study	Not Significant	Not Significant	Significant	Not Significant	Not Significant	Not Significant	Not Significant	Significant	Not Significant

The increased transmutational burden and considerable demonstration of molecules of immune checkpoint could point to elevated immunogenicity in microsatellite instability (MSI) in patients of CRC. There is a rising body of facts hinting that there is an association between tumours having MSI-H phenotype and demonstration of PD-L1 and, thus, anti-PD-1/PD-L1 therapy.

In a study done by Kim et al. CRCs having MSI-H phenotype with positivity of PD-L1 in tumor cells were associated with loss of expression of MLH1/PMS2 and showed significant correlation with older age of onset, female sex, infiltrating growth pattern, advanced AJCC/UICC stage (stage III/IV), lymphovascular invasion, high-density tumor-infiltrating T cells, high-density tumor-infiltrating macrophages, poor differentiation, tumor budding and a lack of mucinous histology. Thus, MSI-H CRCs were significantly associated with early-stage (stage I/II), moderate to marked peritumoral lymphoid reaction, high-density tumor-infiltrating T cells, high-density tumor-infiltrating macrophages (70%), and lack of mucinous histology [17,18]. A study done by Droeser et al. depicted that among the MMR-proficient and MMR- deficient CRC, a strong positivity of PD-L1 was seen. Also, strong PD-L1 expression was associated with early T stage, absence of lymph node metastasis, lower tumor grade, and absence of vascular invasion in MMR-proficient CRC [18].

A study by Wyss et al. showed that PD-L1 expression in tumor cells was significantly associated with tumor stages, whereas the other clinicopathological parameters did not show any significant correlation [19]. A study done by Valentini et al. showed PD-L1 in neoplastic cells was significantly associated with older age, right-sided location, medullary histology, G3 tumor grade, and MSI status, whereas PD-L1 expression in immune cells was significantly associated with right-sided location and MSI [20]. Another study done by Shan et al. showed that the level of PD-L1 expression was significantly associated with stage and TNM stage, lymph node metastasis, and distant metastasis [21].

In our study, PD-L1 expression in the tumor cells showed no significant association with the clinicopathological parameters, whereas PD-L1 expression in immune cells showed a significant association with the gender of patients. PD-L1 positive immune cells were mostly seen in the female gender in 20 (62.5%) cases out of the total 32 cases of females (p-value=0.043). Also, MSI-H, MSI-L, and MSS status showed significant correlation with PD-L1 status (p-value<0.001). Out of the 10 cases which showed MLH1 loss, eight (80%) cases showed positive PD-L1 expression in tumor cells, and out of the 38 cases which showed retained MLH1 expression, 36(94.7%) cases showed retained PDL-1 expression in tumor cells. Of the 11 cases that showed MSI-H instability, nine (81.8%) cases showed positive PD-L1 expression, and out of the seven cases of MSI-L status, all of the cases (100%) showed negative PD-L1 expression. Out of the 37 MSS cases, 29 (78.3%) cases showed negative PD-L1 expression. In contrast, PD-L1 status in immune cells showed no significant correlation with MSI status.

Limitations of the study include a smaller sample size. Also, most of the samples were small colonoscopies biopsies. As a result, tumor heterogeneity could not be limited. PCR was not done. The disparities among various studies show the heterogeneous study population and variable definitions of PD-L1 expression and the use of IHC on core needle biopsies.

## Conclusions

Most of the cases of adenocarcinomas in our study were located in the rectum and had male preponderance. Microsatellite instability with MSI-H status showed a lower rate of distant metastasis and is mostly situated in the proximal colon. PD-L1 status in neoplastic cells showed no significant correlation with any clinicopathological parameters, whereas PD-L1 positivity in immune cells was seen more in the female gender. MSI-H status tumours showed an increased positivity of PD-L1 expression in tumor cells. 

Our study suggests that MSI-H tumors are situated more in the proximal colon and show a lower rate of distant metastasis hence associated with lower M stage. Also, MSI-H status showed increased positivity of PD-L1 in tumor cells which suggests that patients with MSI-H status will benefit from anti-PD-L1 therapy.
